# Putting channels in their place: nucleoporins and symbiotic signaling in legumes

**DOI:** 10.1093/plphys/kiag493

**Published:** 2026-07-11

**Authors:** Neeta Lohani

**Affiliations:** Assistant Features Editor, Plant Physiology, American Society of Plant Biologists; Department of Biotechnology, Thapar Institute of Engineering and Technology, Patiala, Punjab 147004, India

Every cell must decide what enters the nucleus and what stays out. The nuclear pore complex (NPC), built from proteins called nucleoporins (NUPs), spans the double membrane of the nuclear envelope and shuttles molecules between the cytoplasm and the nucleus. Most NPC components serve this universal housekeeping role, so it was striking when a handful turned out to matter specifically for symbiosis. The NUP107-160 subcomplex, which forms the outer ring of the pore (Tamura et al. 2010), was first linked to symbiosis in the model legume *Lotus japonicus*. Mutants in 3 of its members, NUP133, NUP85, and NENA, show impaired root nodulation and arbuscular mycorrhization. These mutants also lack the nuclear calcium spiking triggered by host perception of symbiotic microbes (Kanamori et al. 2006; Saito et al. 2007; Groth et al. 2010). This calcium spiking requires nuclear envelope-localized cation channels, CASTOR and POLLUX, in *Lotus* (Charpentier et al. 2008). The same pathway operates in the related model legume *Medicago truncatula*, where the Pollux ortholog DMI1 fills this role (Ané et al. 2004). The function of these channels likely depends on reaching the inner nuclear membrane, but how these NUPs influence symbiotic signaling has remained unclear for nearly 2 decades.

In this issue of *Plant Physiology*, Kalil et al. (2026) provide an answer. They show that NUP133 is also required for symbiosis in *Medicago* and that the subcomplex controls how DMI1 and POLLUX distribute between the inner and outer nuclear membranes. Overexpressing these channels partially rescues the symbiotic defects, and the degree of rescue shows that nodulation is exquisitely sensitive to how much channel protein reaches the inner membrane.

The authors first asked whether NUP133 has a conserved symbiotic role beyond *Lotus*. They identified a *Medicago* ortholog (*Medtr5g097260.1*) sharing 81% amino acid identity with LjNUP133 and confirmed its nuclear envelope localization by GFP fusion. Overexpression of *MtNUP133* partially restored nodulation in the *Ljnup133* mutant, demonstrating functional equivalence across species. The *Medicago* loss-of-function mutant *Mtnup133* revealed a notably milder phenotype than its *Lotus* counterpart. Nodulation was reduced by roughly one-half rather than nearly abolished, at both 20 °C and 28 °C, and bacteroid differentiation proceeded normally. Arbuscular mycorrhization was also impaired, with reduced root colonization and arbuscule abundance accompanied by a 40% drop in mycorrhiza-dependent shoot biomass. Complementation with *MtNUP133* restored both phenotypes. Unlike in *Ljnup133*, Nod factor–induced calcium spiking still occurred in *Mtnup133* root hairs, although the proportion of responding cells fell by one-half and induction of the early symbiotic markers was significantly reduced. NUP133 is therefore required for robust symbiosis in both legumes, but *Medicago* tolerates its loss far better than *Lotus*.

Why should the same NUP matter so much more in one legume than the other? Confocal microscopy confirmed that POLLUX::GFP and DMI1::GFP localize to the nuclear envelope in both wild-type and mutant backgrounds but could not resolve the inner from the outer membrane. The authors therefore used quantitative immunogold labeling on high-pressure-frozen roots to assign gold particles to one membrane or the other. This approach has well-known technical limits, but a consistent shift across independently analyzed nuclei can reveal genuine biological differences. In wild-type plants, both channels distributed across the inner and outer membranes with a clear preference for the inner one, as previously reported for DMI1 (Capoen et al. 2011). In the *Lotus nup85* and *nup133* mutants, the proportion of particles at the inner membrane dropped by a small but statistically significant 5% to 7%, shifting the balance toward the outer membrane. The *Medicago nup133* mutant showed an even larger shift of 18%, yet displayed only a mild nodulation phenotype. This paradox is central to the study.

The answer to this paradox comes from a dosage experiment. The authors overexpressed CASTOR, POLLUX, or DMI1 in the *Lotus nup133*, *nup85*, and *nena* mutants, reasoning that additional channel protein should compensate for the trafficking defect. CASTOR or POLLUX partially restored nodulation, whereas DMI1 rescued it to much higher levels and produced larger nodules. This hierarchy makes sense given the intrinsic properties of the channels. DMI1 has a longer open time and can functionally substitute for both POLLUX and CASTOR in *Lotus*, but neither POLLUX nor CASTOR can substitute for DMI1 (Venkateshwaran et al. 2012). Because DMI1 is the more efficient channel, the residual inner-membrane pool in *Medicago nup133* remains sufficient to sustain calcium spiking despite the larger redistribution. *Lotus*, by contrast, depends on POLLUX, and even a modest 5% to 7% loss from the inner membrane is enough to drop calcium spiking below the threshold needed for nodulation. Channel identity, not merely the magnitude of mislocalization, dictates the severity of the symbiotic defect.

Together, these results support a model in which the NUP107-160 subcomplex facilitates the transport of DMI1 and POLLUX to the inner nuclear membrane through the central channel of the NPC (Fig. 1). The N-terminal nuclear localization signals and unstructured linker regions of these channels likely guide this transport (Meinema et al. 2011), and their abundance at the inner membrane determines the strength of Nod factor-induced calcium spiking. Loss of specific NUPs leaves the channels stranded at the outer membrane, dampening spiking and, in turn, nodulation and mycorrhization. Several questions remain open. Foremost is the puzzle of specificity. If the outer ring is a single structural unit of more than 10 NUPs, why do only *nup133*, *nup85*, and *nena* disrupt symbiosis? NUP85 and NUP133 form a physically interacting submodule in other eukaryotes and may present a dedicated interface for inner-membrane cargo, possibly through a plant-specific adaptor linking the pore to the symbiosis pathway. It also remains untested whether other nuclear envelope symbiotic proteins, such as the calcium pump MCA8 and the DMI1-associated CNGC15 channels (Charpentier et al. 2016; Cook et al. 2025), depend on the same NUPs. Answering these questions will require systematic interaction mapping among Y-complex members and, ultimately, super-resolution imaging of the plant nuclear envelope.

**Figure 1:**
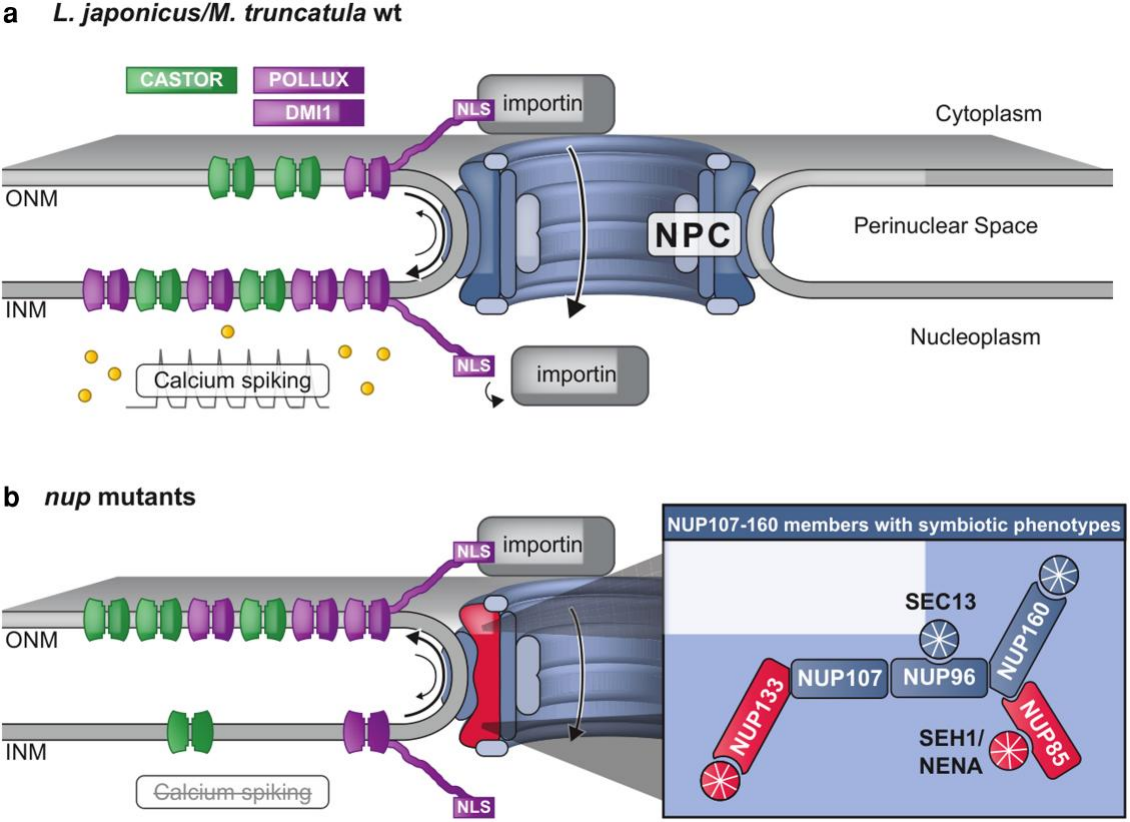
Model of NPC-mediated transport of symbiotic ion channels. a) In wild-type plants, the NUP107-160 subcomplex facilitates transport of CASTOR, POLLUX, and DMI1 from the ONM to the INM, where their accumulation supports nuclear calcium spiking. (b) In NUP mutants, this transport is impaired, and the channels accumulate at the ONM, dampening calcium spiking. NUP107-160 members producing symbiotic phenotypes when mutated are highlighted in red. Adapted from Kalil et al. (2026).

This study carries 2 broader insights. First, the nuclear pore can act as a precision instrument rather than a passive conduit, tuning the spatial distribution of signaling proteins finely enough to alter a whole-plant outcome. Second, because calcium spiking is so sensitive to channel dosage, legume symbiosis offers a powerful system for studying how integral membrane proteins reach the inner nuclear membrane, a process poorly understood even in animal and yeast systems. As efforts to extend nitrogen-fixing symbioses to non-legume crops gather pace, understanding how the pore positions the calcium machinery that licenses these partnerships may prove as important as the signaling components themselves. For these channels, the inner nuclear membrane is not just a destination but a setpoint for symbiotic calcium signaling.

Recent research articles in *Plant Physiology*:

- Kawade et al. (2024) reported that the control of root nodule formation in *Lotus japonicus* is coordinated with shoot water status, linking the systemic regulation of nodule number to whole-plant physiology (https://doi.org/10.1093/plphys/kiae126).
- Xiao et al. (2025) showed that nodule organogenesis in *Medicago truncatula* requires local, stage-specific auxin biosynthesis and transport, underscoring how spatially restricted signaling governs symbiotic organ development (https://doi.org/10.1093/plphys/kiaf133).

## Data Availability

No new data were generated or analyzed in support of this article.
